# Nasopharyngeal carriage of *Staphylococcus aureus* among imprisoned males from Brazil without exposure to healthcare: risk factors and molecular characterization

**DOI:** 10.1186/1476-0711-13-25

**Published:** 2014-07-02

**Authors:** Claudia de Lima Witzel, Carlos Magno Castelo Branco Fortaleza, Camila Sena Martins de Souza, Danilo Flávio Moraes Riboli, Maria de Lourdes Ribeiro de Souza da Cunha

**Affiliations:** 1 Department of Tropical Diseases and Imaging Diagnosis, Faculdade de Medicina de Botucatu, UNESP – Univ Estadual Paulista, Botucatu, SP, Brazil; 2 Department of Microbiology and Immunology, Biosciences Institute, UNESP – Univ Estadual Paulista, Botucatu, SP, Brazil

**Keywords:** *Staphylococcus aureus*, MRSA, Inmates

## Abstract

**Background:**

Previous studies report high prevalence of Methicillin-resistant *Staphylococcus aureus* (MRSA) colonization among imprisoned populations. However, there are no data on that prevalence in Brazilian correctional institutions.

**Findings:**

We tested 302 male prisoners for nasopharyngeal colonization with *Staphylococcus aureus* from February 2009 through April 2010. The overall isolation rate of *S. aureus* was 16.5% (50/302). Men who had sex with men, users of inhalatory drugs and those with previous lung or skin diseases were more likely to be colonized with *S. aureus*. MRSA was isolated from 0.7% of subjects (2/302). The two Community-associated (CA)-MRSA belonged to ST5 but were unrelated based on the PFGE results. Both harbored SCC*mec* IV, and did not possess the Panton-Valentine Leukocidin gene.

**Conclusion:**

We found low prevalence of *S. aureus* and CA-MRSA among prisoners. MRSA isolates ST5 from two subjects harboured SCC*mec* IV and presented different PFGE patterns.

## Background

Methicillin-resistant *Staphylococcus aureus* emerged as relevant agents of community-associated infections in the past decades [1]. Recent studies report that MRSA accounts for as much as 40% of staphylococcal infections in the community settings [2-4]. This picture renewed interest in the epidemiology of *S. aureus* carriage among special populations.

Previous studies emphasize the relevance of MRSA among imprisoned populations [5]. In some prison settings, MRSA may be implicated more that half of skin and soft tissue infections [6,7]. However, there are no data on MRSA in Brazilian correctional institutions. This is a relevant issue, since prison characteristics vary in different countries. Moreover, since those institutions harbor closed populations, it is important to know whether MRSA from those prisons have molecular features similar to those of strains originating in the hospital or in the community. Finally, there is scarce data on CA-MRSA in Brazil. The few reports that included clonal characterization describe strains belonging to the Oceania Southwest Pacific clone (ST30), USA300 (ST8), USA400 (ST1) and USA800 (ST5) [8]. Recently, there seems to be extensive spread of clones belonging to the clonal complex 5 (CC5). One population-based survey found that agent in 0.9% of healthy subjects in the city of Botucatu, São Paulo State [9].

Our study aimed to identify the prevalence of nasopharyngeal colonization with *S. aureus* (including MRSA) among male prisoners form a Brazilian correctional facility. We were especially interested in identifying colonization with CA-MRSA. For that purpose, we assumed the classical definition of CA-MRSA, implying no exposure to health care (absence of previous MRSA colonization, no admission to healthcare setting, long-term facilities, no use of percutaneous devices and no performance of hemodialysis). Coherently, we excluded subjects who could harbour healthcare-associated (HA)-MRSA as a consequence of exposure to hospitals or other healthcare services. Our study also included testing for methicillin resistance with disk diffusion and molecular methods, alongside with identification of genes coding for the Panton-Valentine Leukocidin (PVL) and with molecular typing.

## Methods

The study had a cross-sectional design. It was carried out on the Avaré Center for Re-socialization, in Avaré City, Brazil (23.1**°**S, 48.9**°**W) during the period from February 2009 through April 2010.

We included 302 male prisoners, who signed an informed consent agreeing to participate. All those subjects that had previous exposure to healthcare settings in the past year were excluded. However, since all prisoners underwent regular medical assessment in their own prison environment, we did not exclude subject on the basis of that assessment, provided that no antimicrobials were prescribed.

This study was approved by the reference committee for ethics in research. Nasopharyngeal swabs were obtained from all study subjects. The swabs were manufactured with natural fibers not treated with chemical additives, whitening agents or bleach. The samples were transported in Stuart media (Copan, Italy). Cultures were performed in agar Baird-Parker (Hampshire, UK). Species identification and disk diffusion susceptibility tests (with oxacillin 1 μg and cefoxitin 30 μg) followed standard procedures from the Clinical Laboratory Standards Institute (CLSI) [10]. *S. aureus* ATCC 25923 (oxacillin-sensitive, *mecA* gene negative) and ATCC 33591 (oxacillin-resistant, *mecA* gene positive) were used as controls in all experiments. DNA isolation previous to Polymerase chain reaction (PCR) was performed using Illustra kit (GE Healthcare, Pittsburg, PA), according to the manufacturer’s instructions. PCR was performed for detection of the genes related to methicillin resistance (*mec*A) [11] and to PVL [12]. Methicillin-resistant strains were submitted characterization of the Staphylococcal Chromosome Cassette *mec* (SCC*mec*) [13] and to molecular typing through Pulsed-Field Gel Electrophoresis (PFGE) [14] and Multilocus Sequence Typing (MLST) [15]. Reactants for PCR and PFGE were acquired from the following manufacturers: Taq polymerase, Biotools (Madrid, Spain); PCR primers, Life Technologies (São Paulo, Brazil); *SmaI* restriction enzyme, Fermentas (Pittsburgh, PA). International clones kindly provided by Dr. Antonio Carlos Campos Pignatari, Laboratório Especial de Microbiologia Clínica, Disciplina de Infectologia, Universidade Federal de São Paulo/Escola Paulista de Medicina, and Dr. Agnes Marie Sá Figueiredo, Universidade Federal do Rio de Janeiro, Instituto de Microbiologia Prof. Paulo de Góes, Brazil, were used as controls.

We also analyzed risk factors for colonization with *S. aureus.* Data were obtained though interviews with subjects and review of their medical charts. We recorded demographic information, as well as data on sexual orientation, habits, use of illicit drugs and comorbidities. Statistical analysis was performed in SPSS 19.0 (IBM, Armonk, NY, USA). Data were initially submitted to univariate analysis. Dichotomous data were analyzed using the Chi-square or Fisher Exact test. For continuous variables we used the Student T or Mann-Whitney U test. Subsequently, data were included in multivariable models of logistic regression. A backward selection process for variables was applied, using p-values of 0.05 and 1.0 as requirements for entering and staying in the models, respectively. A final p-value of 0.05 was stated as the limit for statistical significance.

## Findings

**Figure 1:**
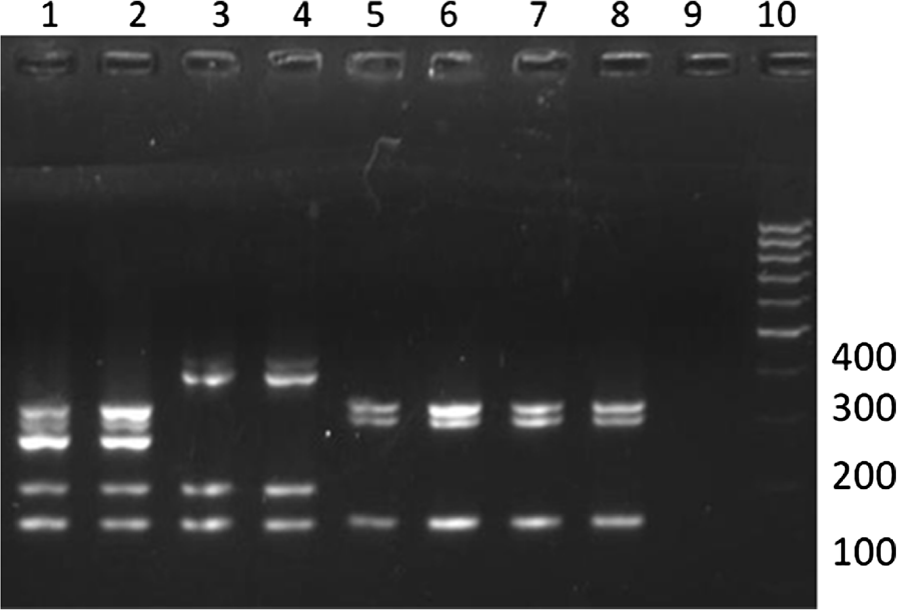
**Agarose gel electrophoresis showing the products amplified by SCC** ***mec *** **multiplex PCR Lane 1, type II (PER34), Lane 2, type II (N315), Lane 3, type III (ANS46), Lane 4, type III (HU25), Lane 5 type IVa (JCSC1968), Lane 6 type IVb (JCSC978/8/6-3P), Lane 7 type IV (Swab nasal 123), Lane 8 type IV (Swab nasal 251), Lane 9, negative control (H**_**2**_**O), Lane 10, DNA molecular size marker (100-bp ladder).**

**Figure 2:**
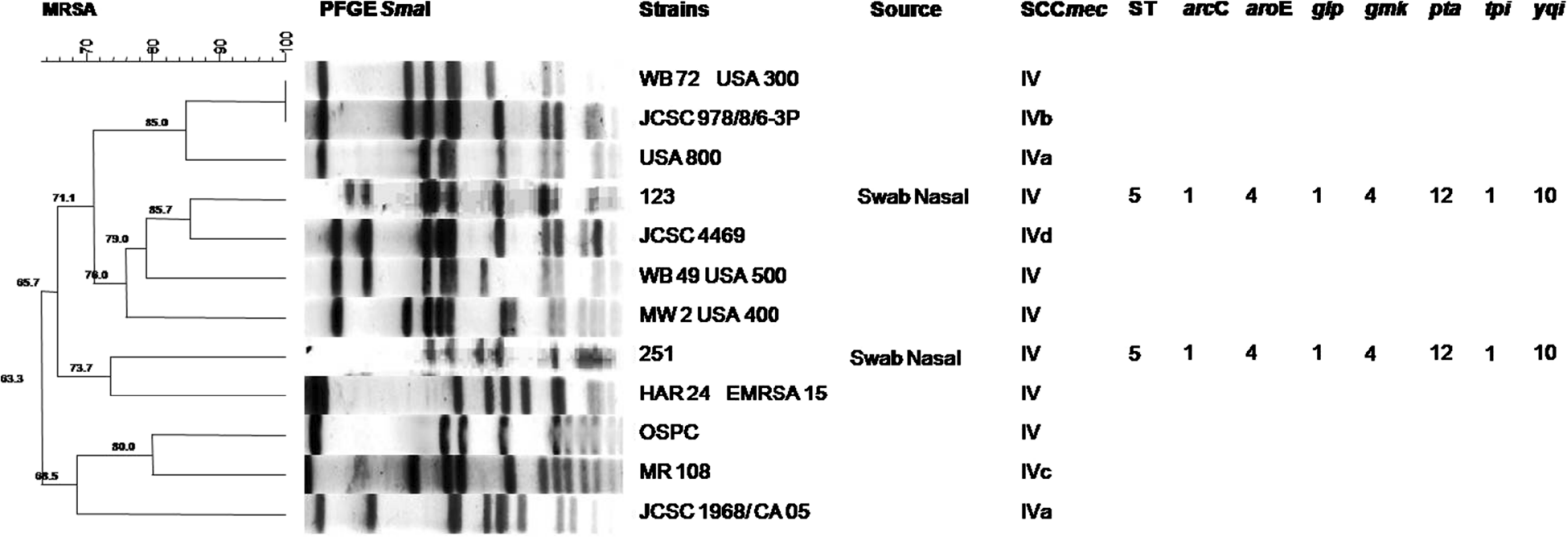
Dendrogram of Pulsed Field Gel Electrophoresis patterns of the two MRSA strains colonizing prison inmates, compared to several international CA-MRSA clones.

Prevalence rates of *S. aureus* and MRSA colonization were 16.5% (95% CI, 12.7%-21.1%) and 0.7% (95% CI, 0.1%-2.2%), respectively. Identification of the two MRSA isolates was confirmed by both phenotypic and genotypic methods. In the disk diffusion tests, methicillin resistance was confirmed according to CLSI breakpoints for both oxacillin and cefoxitin [10]. Also, both isolates harbored the *mecA* gene by the PCR assay. The two MRSA isolates harbored SCC*mec* type IV and belonged to ST5 (Figure 1 and Figure 2). However, each one had a distinct PFGE pattern, ruling out cross-transmission (Figure 2). One of the strains presented 85.7% similarity with the international (Japanese) clone JCSC 4469. Of note, neither those strains nor the methicillin-susceptible isolates from our study tested positive for PVL-coding genes.

Given the low prevalence, it was not possible to analyze risk factors for MRSA colonization. However, we identified independent predictors for colonization with *S. aureus* as a whole: sex with men (Odds Ratio[OR], 3.24; 95% CI, 1.15-8.85; *P* = 0.001), use of inhalatory illicit drugs (OR, 2.24; 95% CI, 1.09-4.59; *P* = 0.008) and recent lung (OR, 6.36; 95% CI, 1.22-33.28; *P* = 0.03) or skin (OR = 2.21; 95% CI, 1.10-4.41; *P =* 0.03) diseases (Table 1).

**Table 1:** **Risk factors for *Staphylococcus aureus *** **colonization among 302 male prisoners in Avaré, Brazil (univariate and multivariable analysis)**

**Risk factors**	**Univariate analysis**				**Multivariable analysis**	
	***S. aureus *** **positive (n = 50)**	***S. aureus *** **negative (n = 252)**	**OR (95% CI)**		**OR (95% CI)**	***P***
*Demographic data*						
Age, mean	32.8	32.9	…	0.95		
White (race)	**40 (80.0)**	**184 (73.0)**	**1.48 (0.70-3.12)**	**0.03**		
Complete fundamental schooling*	11 (22.0)	52 (20.6)	1.09 (0.52-2.26)	0.83		
Marriage or stable relationship	31 (62.0)	146 (57.1)	1.19 (0.64-2.21)	0.30		
Body mass index, mean	23.6	23.1	…	0.64		
*Behavioral factors*						
Sex with men	**9 (18.0)**	**16 (6.3)**	**3.24 (1.34-7.82)**	**0.001**	**3.20 (1.15-8.85)**	**0.03**
Use of illicit drugs (injectable)	0 (0.0)	11 (4.4)	0.0 (indefinite)	0.22		
Use of illicit drugs (inhalatory)**	**27 (54.0)**	**91 (36.1)**	**2.08 (1.13-3.83)**	**0.02**	**2.24 (1.09-4.59)**	**0.03**
Alcoholism	29 (58.0)	155 (61.5)	0.86 (0.47-1.60)	0.64		
Smoke	37 (74.0)	189 (75.0)	0.95 (0.48-1.89)	0.88		
Regular practice of sports	7 (14.0)	34 (13.5)	1.04 (0.43-2.50)	0.92		
Works while in prison	34 (68.0)	146 (57.9)	1.54 (0.81-2.94)	0.18		
Tatoo	26 (52.0)	148 (58.7)	0.77 (0.41-1.40)	0.38		
Tatto in the past six months	13 (26.0)	31 (32.1)	0.74 (0.37-1.47)	0.39		
*Characteristics of prison regime*						
Months in prison, median (range)	9.5 (0-62)	11 (1-96)	…	0.22		
Closed regime	44 (88.0)	212 (84.5)	1.62 (0.60-4.34)	0.33		
Regular intimal visits***	25 (50.0)	106 (46.8)	1.38 (0.75-2.53)	0.30		
*Comorbidities*						
Heart disease	7 (14.0)	36 (14.3)	0.98 (0.40-2.33)	0.96		
Lung disease	**4 (8.0)**	**5 (2.0)**	**4.20 (1.11-16.60)**	**0.04**	**6.36 (1.22-33.28)**	**0.03**
Liver disease	2 (4.0)	5 (1.2)	3.43 (0.56-21.08)	0.20		
Skin disease	**26 (52.0)**	**83 (33.1)**	**2.29 (1.23-4.25)**	**0.008**	**2.21 (1.10-4.41)**	**0.03**
Diabetes mellitus	1 (2.0)	4 (1.6)	1.26 (0.14-11.47)	1.0		
Seropositive for HIV	0 (0.0)	2 (0.8)	0.0 (indefinite)	1.0		
Hepatitis B	1 (2.0)	4 (1.6)	1.26 (0.14-11.47)	1.0		
Hepatitis C	1 (2.0)	2 (0.8)	2.59 (0.23-29.18)	0.92		
Syphillis	1 (2.0)	5 (2.0)	1.01 (0.12-8.82)	1.0		

Note. Data are in number (%), unless otherwise specified. CI, Confidence Interval; OR, Odds Ratio. Statistically significant results presented in boldface.

*In Brazil, fundamental schooling means 8 years in school.

**Marijuana, cocaine or crack.

***Visits from wife or other sexual partner are allowed.

## Discussion

Presently there are 494 thousand people living in correctional institutions in Brazil - the world’s 3^rd^ greater prison population (data from Brazil’s “Conselho Nacional de Justiça” [National Council of Justice], available at http://www.cnj.jus.br) [16]. Therefore, the potential dissemination of MRSA in Brazilian prisons is a matter of special concern.

Interestingly, the prevalence of MRSA colonization in our study was similar to that reported in population-based surveys [17]. Our finding contrasts with those from previous studies that report at least 1.8 times greater prevalence of MRSA carriage among prisoners, as compared to the general population [18]. One recent study conducted in maximum-security prisons from the United Sates found MRSA carriage among 5.9% of inprisoned males [19]. A study form Ethiopia found MRSA in nasal swabs from 12 out of 50 healthy prisoners [20]. Overcrowding, use of illicit drugs and even environmental contamination have been implicated in the extensive cross transmission of MRSA in prisons [18,21].

Even more surprising was the lower prevalence of *S. aureus* and MRSA carriage among prisoners, when compared to results from a general population-based survey conducted in the same region of Brazil. In that study, 32.7% and 0.9% of persons carried *S. aureus* and MRSA, respectively [9]. Even though these findings are somewhat puzzling, some remarks should be made regarding the study subjects.

First of all, one should notice that both the general population and the prison studies were conducted simultaneously in the same research laboratory, applying the same techniques and protocols. Thus, we can rule out methodological disparities. Other important detail is that a significant proportion of the carriers in the population-based survey were aged less than 18 years. That is the threshold for penal adulthood in Brazil, so all the prisoners belonged to higher age groups, though most were young adults.

There are many different kinds of correctional institutions in Brazil. Most of them present characteristics that are usually associated with greater likelihood of *S. aureus* and/or MRSA spread: overcrowding, poor sanitation and high prevalence of HIV infection and tuberculosis [22]. In that sense, Avaré Center for Ressocialization has atypical features. In the last few years, several improvements in disease prevention (e.g., vaccination and safe sex campaigns) were undertaken. Also, it is a prison for minor crimes, with wide cells and broad outdoor space. There are strict rules for personal hygiene. Daily baths, shaven beards and short hair are mandatory. All prisoners are vaccinated (e.g., against tetanus and hepatitis B), submitted to regular medical assessment in the prison. The prisoners must attend lectures on diseases prevention. The HIV prevalence in the prison is low, and no prisoner with tuberculosis was admitted during the study period. Still, the use of illicit drugs is common, in spite of restriction policies. That habit was associated with greater likelihood of carrying *S. aureus* in our study. Sex with men, a common risk factor for infection with community-associated MRSA (CA-MRSA) [23], was a predictor of overall *S. aureus* colonization. It is worth noting that one of the two MRSA carriers reported frequent sexual intercourse with other men.

The two MRSA strains, though unrelated on PFGE, harbored SCC*mec* type IV. Both belonged to ST5, which seems to be widespread in Brazil [9,24]. This points out to acquisition of strains from the community – rather than spread from subjects with exposure to healthcare. However, though previous reports found association between SCC*mec* IV-harboring clones and PVL production, not a single *S. aureus* strain from our study tested positive for PVL-related genes. Since PVL is related to severe skin diseases [12], this finding is coherent with the lack of reports of that condition among the study population. Rodriguez-Noriega et al. [24] state that CA-MRSA clones have been detected in Brazil at increasing rate. This is particularly true for strains related to the pediatric clone. That clone belongs to CC5, which is widespread in Latin America and ranks among the five greatest clonal complexes causing infections worldwide. Studies of single nucleotide polymorphisms (SNP) of ST5-MRSA isolates suggest that their spread is due to several episodes of SCC*mec* insertions into various methicillin-susceptible *S. aureus* strains in different regions of the world, not to the global spread of a single ST5-MRSA clone [25]. Even though our study found only two subjects carrying CA-MRSA, molecular typing of the isolates is coherent with regional epidemiological data.

## Conclusion

To date, outbreaks of CA-MRSA among Brazilian prisoners were not reported. Given the wide variation of sanitary condition and population among prisons in Brazil, we can only guess that prevalence of MRSA colonization and infection may be higher in other correctional facilities. Our study is a first step, one that should be followed by further research, enrolling several different institutions.

## Availability of supporting data

The data set supporting the results of this article is included within the article.

## Abbreviations

MRSA: Methicillin-resistant *Staphylococcus aureus*; CA-MRSA: Community-associated methicillin-resistant *Staphylococcus aureus*; ST: Sequence type; PFGE: Pulsed-field gel electrophoresis; PVL: Panton-valentine leukocidin; SCC*mec*: Staphylococcal chromosome cassette *mec*; CC: Clonal complex; HA-MRSA: Healthcare-associated Methicillin-resistant *Staphylococcus aureus*; PCR: Polymerase chain reaction; *mec*A: Methicillin resistance gene; MLST: Multilocus Sequence Typing; CLSI: Clinical laboratory standards institute.

## Competing interests

All authors declare they have no competing interests regarding the present study.

## Authors’ contributions

CLW: participated in the conception and design of the study, carried out the microbiological tests, and participated in the writing of the paper. CMCBF: participated in the conception and design of the study, analyzed the clinical data, performed the statistical analysis and wrote the paper. CSMS: helped with the microbiological tests and performed the MLST. DFMR: performed the PFGE and analyzed the data. MLRSC: responsible for the conception and design of the study, coordination of laboratory work, data analysis and manuscript writing. All authors read and approved the final manuscript.
